# Engineering human/simian rotavirus VP7 reassortants in the absence of UTR sequence information

**DOI:** 10.1007/s00253-025-13435-z

**Published:** 2025-02-27

**Authors:** Roman Valusenko-Mehrkens, Reimar Johne, Alexander Falkenhagen

**Affiliations:** Department of Biological Safety, German Federal Institute for Risk Assessment, Max-Dohrn-Str. 8-10, 10589 Berlin, Germany

**Keywords:** Rotavirus A, Reverse Genetics, Reassortment, VP7, Untranslated Regions, Point Mutation

## Abstract

**Abstract:**

Recently developed plasmid-based reverse genetics systems for rotavirus A (RVA) enable rapid engineering of reassortants carrying human RVA antigens. However, complete genome segment sequences are required for successful generation of such reassortants, and sequencing of the untranslated regions (UTRs) of field strains is often not accomplished. To address this problem, we established a system that permits the generation of reassortants using only the open reading frame (ORF) nucleotide sequence information. Plasmids containing the VP7-ORF nucleotide sequence of six human RVA field strains (genotypes G2, G5, G8, G9, G12 and G29) derived from GenBank and flanked by the UTR sequences of simian RVA strain SA11 were constructed. Using these plasmids, four VP7 (G2, G5, G9 and G12) reassortants in an SA11 backbone were successfully generated. In contrast, the G8 and G29 reassortants were not viable. BLASTp search of the G8 and G29 sequences revealed an unusual amino acid substitution in each sequence, which was not present in related field strains. Site-directed reversion of the corresponding C656T mutation in G8 led to effective rescue of reassortant virus. However, reverting the G84C mutation in G29 did not result in replicating virus. The results suggest that most human RVA VP7 UTRs can be substituted with simian RVA UTRs. However, generation of reassortants might be impeded by potential sequencing errors or intrinsic reassortment limitations. The established system could help to broaden the antigenic repertoire for rapid engineering of potential novel RVA vaccine strains.

**Key Points:**

*• Generation of diverse rotavirus vaccine strains is impeded by missing UTR sequences.*

*• UTRs from SA11 can be used instead of missing UTR sequences from field strains.*

*• Human RVA reassortants of genotypes G2, G5, G8, G9, G12 were successfully rescued.*

**Supplementary Information:**

The online version contains supplementary material available at 10.1007/s00253-025-13435-z.

## Introduction

Rotavirus A (RVA; species *Rotavirus alphagastroenteritidis*)(ICTV 2024) belongs to the family *Sedoreoviridae* (Matthijnssens et al. 2022) and is the leading cause of severe gastroenteritis requiring hospitalization in infants and young children worldwide (Cohen et al. 2022). The RVA genome is distributed across eleven different double-stranded (ds) RNA segments, which can reassort when a cell is simultaneously infected with different RVA strains (McDonald et al. 2016). Each genome segment consists of a 5’ untranslated region (UTR), one or two open reading frames (ORFs), and a 3’ UTR (Matthijnssens et al. 2022). The UTRs play important regulatory roles in viral genome replication and gene expression (Gratia et al. 2016). The first nucleotides of the 5’ UTR and the last nucleotides of the 3’ UTR are highly conserved across the eleven RVA genome segments and different RVA strains (Uprety et al. 2021). However, the UTR nucleotides between the conserved regions and the ORFs vary in length and composition across different genome segments and RVA strains. The ORFs encode five to six non-structural proteins (NSP1-NSP6) and six structural proteins (VP1, VP2, VP3, VP4, VP6 and VP7). VP4 and VP7 are exposed on the virion surface, known to elicit neutralizing antibody responses and are remarkably diverse (Desselberger 2014) with 58 VP4 genotypes (referred to as P[1–58]) and 42 VP7 genotypes (referred to as G1-42) being known so far (RCWG 2024).

The widespread use of the two attenuated RVA vaccines Rotarix and RotaTeq has reduced mortality due to RVA infection on a global scale. Rotarix is based on a human RVA G1P[8] strain and RotaTeq on five different bovine-human RVA reassortants that have human RVA VP4 P[8] as well as VP7 G1, G2, G3 and G4 (Heaton et al. 2005; Vesikari et al. 2009; Ward and Bernstein 2009). Both vaccines had an efficacy between 85–98% in high-income countries, but a reduced efficacy of 50–64% in low-income countries in Africa and Asia (Desselberger 2017; Jonesteller et al. 2017). The reasons for this discrepancy are not fully understood, but it was proposed that, among other factors, a higher variability in circulating RVA strains could be responsible for the lower vaccine efficacy (Patton 2012; Todd et al. 2010).

The development of new vaccines based on RVA strains with a different immunogenic profile is impeded by the poor replication of clinically relevant human RVA strains in cell culture. However, the recent development of plasmid-based reverse genetics systems facilitated the generation of recombinant rotaviruses with human RVA antigens (Kanai et al. 2017; Komoto et al. 2019). Using such reverse genetics approaches, human-simian reassortants with human RVA VP7 G1, G2, G3, G4, G8, G9, G12 and G16 have already been generated by multiple research groups (Falkenhagen et al. 2020; Fukuda et al. 2020; Kanai et al. 2020; Kawagishi et al. 2020; Sanchez-Tacuba et al. 2020; Valusenko-Mehrkens et al. 2023). However, one drawback of this approach is that complete genome segment sequence information is required. Especially sequencing of the complete UTRs is technically challenging and often not accomplished. Therefore, only coding sequences without complete UTR sequences are mostly available for RVA genome segments in sequence databases.

In the current study, we investigated whether we are able to generate human-simian reassortants if only the human RVA ORF nucleotide sequence, but not its complete UTR sequence, is available. To that end, we constructed genome segment chimeras, which were comprised of a human RVA VP7-encoding sequence flanked by simian RVA UTRs. Using a plasmid-based reverse genetics approach, we could show that the UTRs can be substituted with the corresponding UTRs from simian RVA, but generation of reassortants can still be impeded by potential sequencing errors or reassortment limitations. These insights could help to broaden the antigenic repertoire for potential novel RVA vaccine strains.

## Materials and methods

### Cell lines and viruses

The fetal hamster kidney-derived cell line BSR-T7/5 (Buchholz et al. 1999) was a kind gift from Dr. Karsten Tischer (Free University of Berlin, Berlin, Germany). The fetal African green monkey kidney-derived cell line MA-104 (Whitaker and Hayward 1985) was supplied by The European Collection of Authenticated Cell Cultures (Salisbury, UK). Both cell lines were cultured as described in detail previously (Falkenhagen et al. 2019). Table 1 lists the virus strains used in this study, their abbreviations, and the GenBank accession number for the VP7-encoding genome segment. Recombinant SA11 (rSA11) and all reassortants were generated by using the plasmid-based reverse genetics system described below.

**Table 1:** RVA strains used in this study

Strain	Abbreviation	Genome segment 9 acc. no	Reference
RVA/Simian-tc/ZAF/SA11-L2/1958/G3P[2]	SA11	LC178569	(Kanai et al. 2017)
RVA/Human-wt/MOZ/0060a/2012/G12P[8]	Moz60a	MG926763	(Strydom et al. 2019)
RVA/Human-wt/MOZ/0308/2012/G2P[4]	Moz308	MG926730	(Strydom et al. 2019)
RVA/Human-wt/ZAF/GR10924/1999/G9P[6]	GR10924	FJ183360	(Potgieter et al. 2009)
RVA/Human-wt/ZMB/UFS-NGS-MRC-DPRU4723/2014/G5P[6]	Zmb5	MT271025	(Maringa et al. 2020)
RVA/Human-wt/UGA/MUL-13-308/2013/G8P[6]	Uga8	KX632259	(Bwogi et al. 2017)
RVA/Human-wt/CAF/CAR91/2014/G29P[6]	Caf29	MT163234	(Banga-Mingo et al. 2021)

### Plasmids

The plasmids pT7/VP1SA11, pT7/VP2SA11, pT7/VP3SA11, pT7/VP4SA11, pT7/VP6SA11, pT7/VP7SA11, pT7/NSP1SA11, pT7/NSP2SA11, pT7/NSP3SA11, pT7/NSP4SA11, pT7/NSP5SA11, pCAG-D1R, pCAG-D12L and pCAG-FAST-p10 (Kanai et al. 2017) were a kind gift from Dr. Takeshi Kobayashi and supplied by Addgene (Watertown, MA, USA). The generation of the plasmid encoding the complete VP7-encoding genome segment of Moz60a has previously been described (Falkenhagen et al. 2020). For the generation of the plasmids containing a chimeric genome segment comprised of a human RVA ORF with simian RVA UTRs, entire expression cassettes flanked by restriction sites were synthesized by Integrated DNA Technologies (IDT, Coralville, IA, USA) as dsDNA fragments and cloned directly into compatible restriction sites of the vector pUC-IDT-Amp (IDT) using standard cloning techniques. The expression cassettes were identical to the expression cassette of the pT7-VP4(02V0002G3) plasmid (GenBank: KT239165) (Trojnar et al. 2013), but contained the VP7-encoding ORF of Moz60a, Moz308, GR10924, Zmb5, Uga8 or Caf29 flanked by UTRs of the SA11 VP7-encoding genome segment instead of the complete VP4-encoding genome segment. The Phusion Site-Directed Mutagenesis Kit (Thermo Fisher Scientific, Waltham, MA, USA) was utilized as described previously (Valusenko-Mehrkens et al. 2024) to introduce specific point mutations into the plasmids encoding VP7 from human RVA strains Caf29 and Uga8. 5′-phosphorylated primers used for mutagenesis are listed in Supplementary Table S1. The sequences of the expression cassettes of all plasmids were verified by Sanger sequencing (Eurofins Genomics GmbH, Ebersberg, Germany) using the sequencing primers shown in Supplementary Table S1. Plasmids used for transfection experiments were purified with the QIAfilter Plasmid Midi Kit (Qiagen GmbH, Hilden, Germany).

### Plasmid-based reverse genetics system and passaging

The plasmid-based reverse genetics system and passaging were performed as described in detail previously (Valusenko-Mehrkens et al. 2023). In brief, BSR-T7/5 cells were transfected with the plasmids containing the expression cassettes for the rotavirus genome segments and three helper plasmids. Following transfection, the cells were co-cultured with MA-104 cells. The co-cultured cells including the culture media were lysed by freezing and thawing and clarified lysates were used to inoculate MA-104 cells. Following incubation for seven days, the inoculated MA-104 cells including the culture media were lysed by freezing and thawing and clarified lysates were used for serial passaging on MA-104 cells under the same conditions.

### RNA extraction and analyses by quantitative RT-PCR or RT-PCR

RNA extraction from clarified lysates, DNase digests and quantitative RT-PCRs (qRT-PCRs) were performed as described previously (Valusenko-Mehrkens et al. 2023). For the confirmation of the identity of the generated viruses, viral RNA was amplified by RT-PCR using the OneStep RT-PCR Kit (Qiagen, Hilden, Germany) with primers listed in Supplementary Table S1. Thereafter, the PCR products were cleaned up as described previously (Valusenko-Mehrkens et al. 2024) and Sanger sequencing was performed by a commercial company (Eurofins Genomics GmbH) with the primers used for RT-PCR.

### Plaque assay and analyses of replication kinetics

Plaque assays and analyses of replication kinetics were performed as described previously (Valusenko-Mehrkens et al. 2023). In brief, confluent MA-104 cells were infected with 1.5 × 10^3^ plaque forming units (PFU) of the indicated virus. The number of PFU/ml present in the culture supernatant of infected cells was determined by plaque assay on MA-104 cells at the indicated time points.

### Sequence alignments and protein structure analyses

MegAlign Pro (DNASTAR Inc., Madison, WI, USA) was used for nucleotide sequence alignments. Protein structure analyses, predictions and visualizations were performed using UCSF Chimera (University of California, San Francisco, CA, USA)(Pettersen et al. 2004) based on the atomic model of an infectious rotavirus particle (PDB 4v7q)(Settembre et al. 2011).

### Statistics

Data are means ± standard deviation (SD). Statistically significant differences (*p* ≤ 0.05) were identified utilizing a two-tailed unpaired *t* test.

## Results

### Generation of reassortants using the VP7-encoding region of human rotavirus and the UTRs of simian rotavirus

**Fig. 1:**
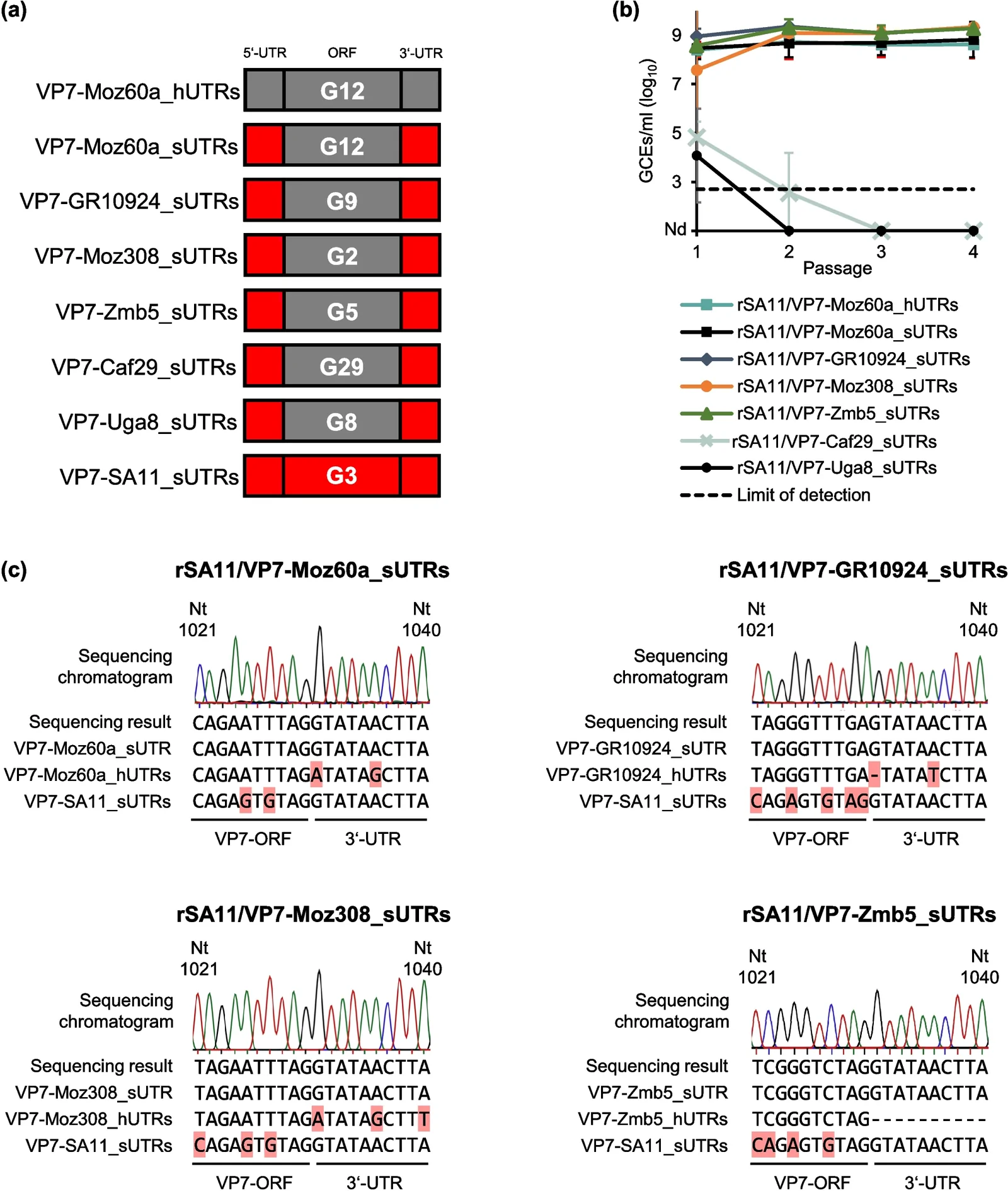
Generation of reassortants with the VP7-encoding ORF of human RVAs flanked by simian UTRs **(a)** Schematic illustration of the VP7-encoding genome segments used for rescue experiments. The VP7 genotype is indicated inside the ORF-bar. Grey = Human RVA fragment; red = SA11 fragment. **(b)** Analysis of the clarified lysates by qRT-PCR after the indicated passages in MA-104 cells. Data are means ± standard deviation from two independent experiments performed in duplicates. **(c)** Sanger sequencing analyses of the VP7-encoding genome segment from the successfully generated reassortants. Respective sequencing chromatograms of passage 4 viruses are shown. A peak in the chromatogram indicates the corresponding nucleotide in the DNA sequence (black = guanine, blue = cytosine, green = adenine, red = thymine). The 3’ end of the ORF and the first 10 bases of the 3’ UTR are depicted. The alignments below the chromatograms compare the sequencing result to the expected chimeric sequence, the corresponding human RVA sequence, and SA11 (nucleotides 1021–1040). The red squares mark differences from the sequencing result. The 3’ UTR of Zmb5 is unknown. Abbreviations: rSA11/VP7-Moz60a_hUTRs, rSA11/VP7-GR10924_hUTRs, rSA11/VP7-Moz308_hUTRs, and rSA11/VP7-Zmb5_hUTRs = Reassortants with VP7-ORF and original UTRs from the corresponding human RVA strain in the backbone of SA11; rSA11/VP7-Moz60a_sUTRs, rSA11/VP7-GR10924_sUTRs, rSA11/VP7-Moz308_sUTRs, rSA11/VP7-Zmb5_sUTRs, rSA11/VP7-Caf29_sUTRs, rSA11/VP7-Uga8_sUTRs = Reassortants with the VP7-ORF of the indicated human RVA strain flanked by SA11 UTRs in the backbone of SA11; UTRs = Untranslated regions; GCEs = Genome copy equivalents; Nd = not detected; Nt = nucleotide

We have previously shown that reassortants carrying the complete VP7-encoding genome segment from African human RVA strains Moz60a (G12), Moz308 (G2) and GR10924 (G9) in a simian RVA strain SA11 backbone were viable (Falkenhagen et al. 2020). These African human RVA strains have never been adapted to cell culture and were chosen because they represented genotypes circulating in Africa for which complete genome segment sequence data were available. To test if the human RVA UTRs can be substituted by the corresponding simian RVA UTRs, plasmids containing a chimeric VP7-encoding genome segment with the ORF from Moz60a, Moz308 or GR10924 flanked by UTRs from SA11 were constructed. Additionally, three plasmids containing the VP7-ORF of human RVA strains Zmb5 (G5), Uga8 (G8) or Caf29 (G29) flanked by SA11 UTRs were constructed. These strains represented uncommon genotypes that circulated in Africa, have also never been adapted to cell culture, and it was unknown if their VP7-encoding genome segment was compatible with simian RVA SA11. For Uga8, the complete UTR sequences were reported, but for Zmb5 and Caf29 only ORF sequence data were publicly available. An overview of all constructs used in this study is shown in Fig. 1a. Sequence alignments of the 5’ and 3’ UTR of the four human RVA strains with known UTRs and simian RVA strain SA11 are shown in Supplementary Fig. S1 and S2, respectively.

Using a plasmid-based reverse genetics system, the generation of viable reassortants was tested by transfection of the newly constructed chimeric VP7-encoding plasmids together with the other 10 genome segments from SA11 as well as three helper plasmids. The plasmid containing the complete Moz60a VP7-encoding genome segment with the original UTRs served as a positive control. BSR-T7/5 cells were transfected and then co-cultured with MA-104 cells. Generated virus was then serially passaged on MA-104 cells until passage number 4. RNA was extracted from clarified lysates after each passage and analyzed by qRT-PCR using RVA NSP3-specific probe and primers (Fig. 1b). RVA RNA was detected for the reassortant containing the VP7-ORF of Moz60a flanked by human RVA UTRs as well as for the reassortants with the VP7-ORF of Moz60a, Moz308, GR10924 or Zmb5 flanked by SA11 UTRs after passage 1 until the end of passage 4, showing that replicating viruses were generated. In contrast, no viral RNA could be detected for the reassortants with the VP7-ORF of Caf29 or Uga8 flanked by SA11 UTRs after the third and fourth passage, indicating that no viable reassortants were generated.

RNA obtained from clarified lysates after passage 4 was further analyzed by RT-PCR followed by Sanger sequencing using primer pairs targeting the 3’-terminal regions of the different VP7-encoding genome segments of the generated reassortants. By this, the expected sequence could be confirmed for each generated reassortant (Fig. 1c). These results showed that, in principle, the UTRs of the VP7-encoding genome segment of human RVAs can be substituted with the ones of simian strain SA11. However, some limitations seemed to exist as we were unable to rescue two of the reassortants.

### Testing the significance of identified point mutations in strains Uga8 and Caf29

In order to determine if VP7 of the two reassortants that we were unable to generate contained unusual amino acid residues, the National Center for Biotechnology Information (NCBI) database of protein sequences was screened using the NCBI Protein Basic Local Alignment Search Tool (BLASTp) (Altschul et al. 1997) with the complete VP7 amino acid sequence of Uga8 or Caf29 as input. The search revealed two amino acid substitutions, one in each sequence, which were not present in related field strains and resulted in amino acid residues with different physico-chemical properties. VP7 of Uga8 contained valine (Val) at position 219, while the 100 most related sequences had the smaller alanine (Ala) at the same position. Supplementary Table S2 shows the five RVA strains with the most related VP7 amino acid sequence and their amino acid residues from position 211 to position 230 in comparison to Uga8. VP7 of Caf29 had asparagine (Asn) at position 28. In contrast, the positively charged lysine (Lys) or arginine (Arg) were present at position 28 of the 100 most related sequences, whereby the four closest hits had Lys (Supplementary Table S3). It is of note that two additional unique amino acid substitutions in the Caf29 sequence resulting in amino acid residues with similar properties were also identified by BLASTp. Serine (Ser) at position 68 of Caf29 VP7 was not found in the other 100 related strains. Instead, all but two of the 100 most related strains had Ala. In addition, threonine (Thr) at position 73 was only present in the Caf29 sequence. However, the amino acid residue at position 73 of the 100 closest related sequences was highly variable. Supplementary Table S4 shows all identified unique amino acid residues for Uga8 and Caf29 as well as the corresponding residues in the 100 closest related strains.

**Fig. 2:**
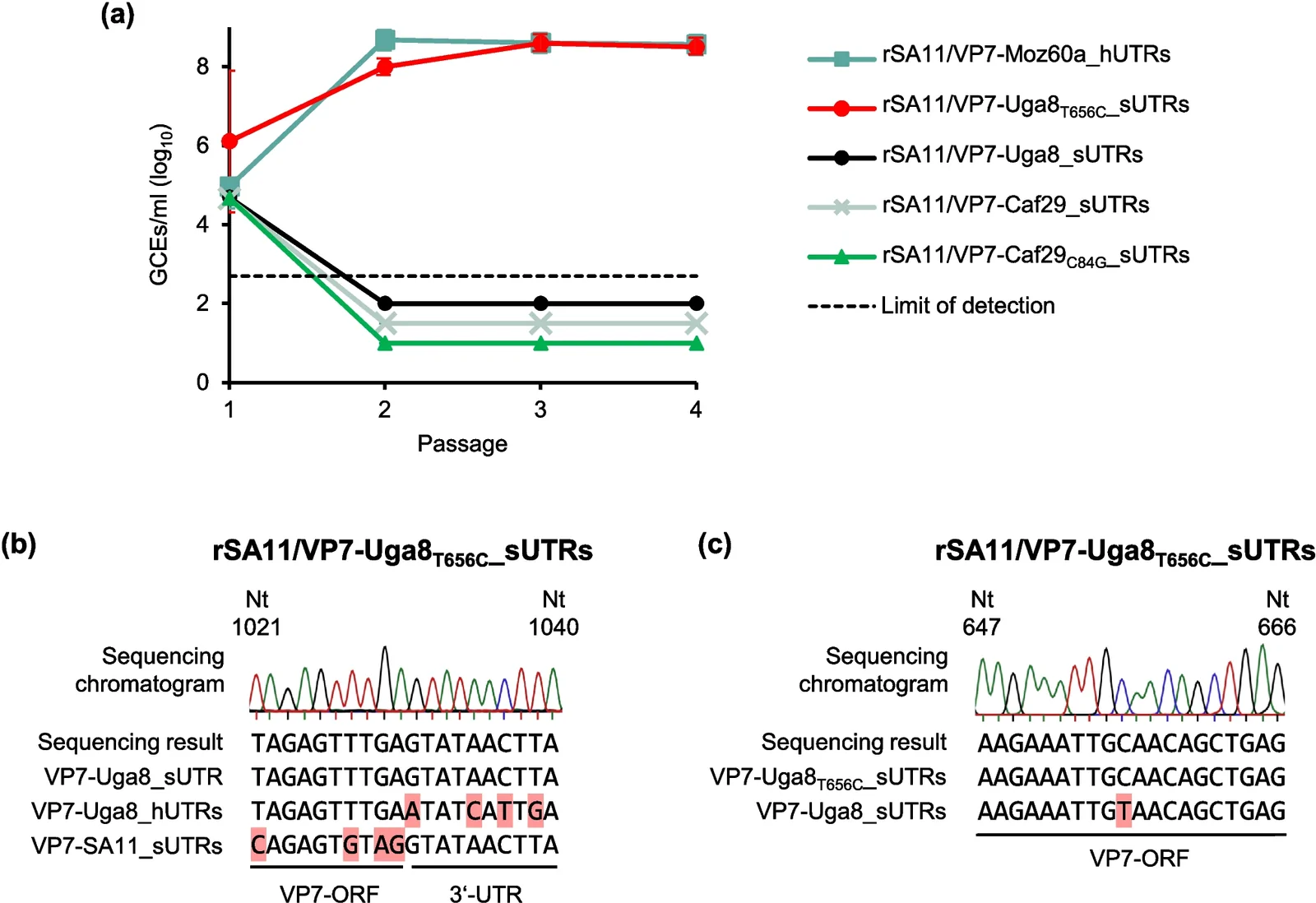
Generation of Uga8 and Caf29 reassortants containing mutation T656C or C84G, respectively **(a)** Analysis of the clarified lysates by qRT-PCR after the indicated passage number in MA-104 cells. Data are means ± standard deviation from two independent experiments performed in duplicates. **(b-c)** Sanger sequencing analyses of the VP7-encoding genome segment from the reassortant containing the Uga8 mutation T656C. Sequencing chromatograms of passage 4 virus are shown. A peak in the chromatogram indicates the corresponding nucleotide in the DNA sequence (black = guanine, blue = cytosine, green = adenine, red = thymine). **(b)** Sequencing of the 3’ end of the ORF and the first 10 bases of the 3’-UTR. The alignment under the chromatogram compares the sequencing result to the expected chimeric sequence, the corresponding human RVA sequence, and SA11 (nucleotides 1021–1040). The red squares mark differences from the sequencing result. **(c)** Sequencing of the ORF region containing the T656C mutation. The alignment under the chromatogram is a comparison between the sequencing result and the Uga8 sequences with or without mutation T656C (nucleotides 652–660). The red square marks the difference from the sequencing result. Abbreviations: rSA11/VP7-Moz60a_hUTRs, rSA11/VP7-Uga8_hUTRs = Reassortants with VP7-ORF and original UTRs from the indicated human RVA strain in the backbone of SA11; rSA11/VP7-Uga8_sUTRs, rSA11/VP7-Uga8_T656C__sUTRs, rSA11/VP7-Caf29_sUTRs, rSA11/VP7-Caf29_C84G__sUTRs = Reassortants with the VP7-ORF of the indicated human RVA strain with or without the indicated mutation flanked by SA11 UTRs in the backbone of SA11; UTRs = Untranslated regions; GCEs = Genome copy equivalents; Nt = nucleotide

To substitute the identified unusual amino acid residues that had different physico-chemical properties with residues that were present in related field strains, we utilized site-directed mutagenesis to introduce a T656C point mutation into the Uga8 VP7-ORF (Uga8_T656C_) of the corresponding plasmid leading to V219A. Similarly, mutation C84G was introduced into the plasmid encoding VP7 of Caf29 (Caf29_C84G_) resulting in N28K. Experiments to generate reassortants with or without the respective mutations were performed as described above. Analyses of passages by qRT-PCR indicated that introducing V219A into VP7 of Uga8 resulted in the successful generation of a reassortant, while N28K in VP7 of Caf29 did not facilitate rescue (Fig. 2a). RNA of the newly generated reassortant was analyzed by RT-PCR followed by Sanger sequencing, which confirmed the presence of the expected chimeric genome segments (Fig. 2b) and the mutation in Uga8_T656C_ (Fig. 2c).

### Characterization of selected reassortants by growth curves and plaque size

**Fig. 3:**
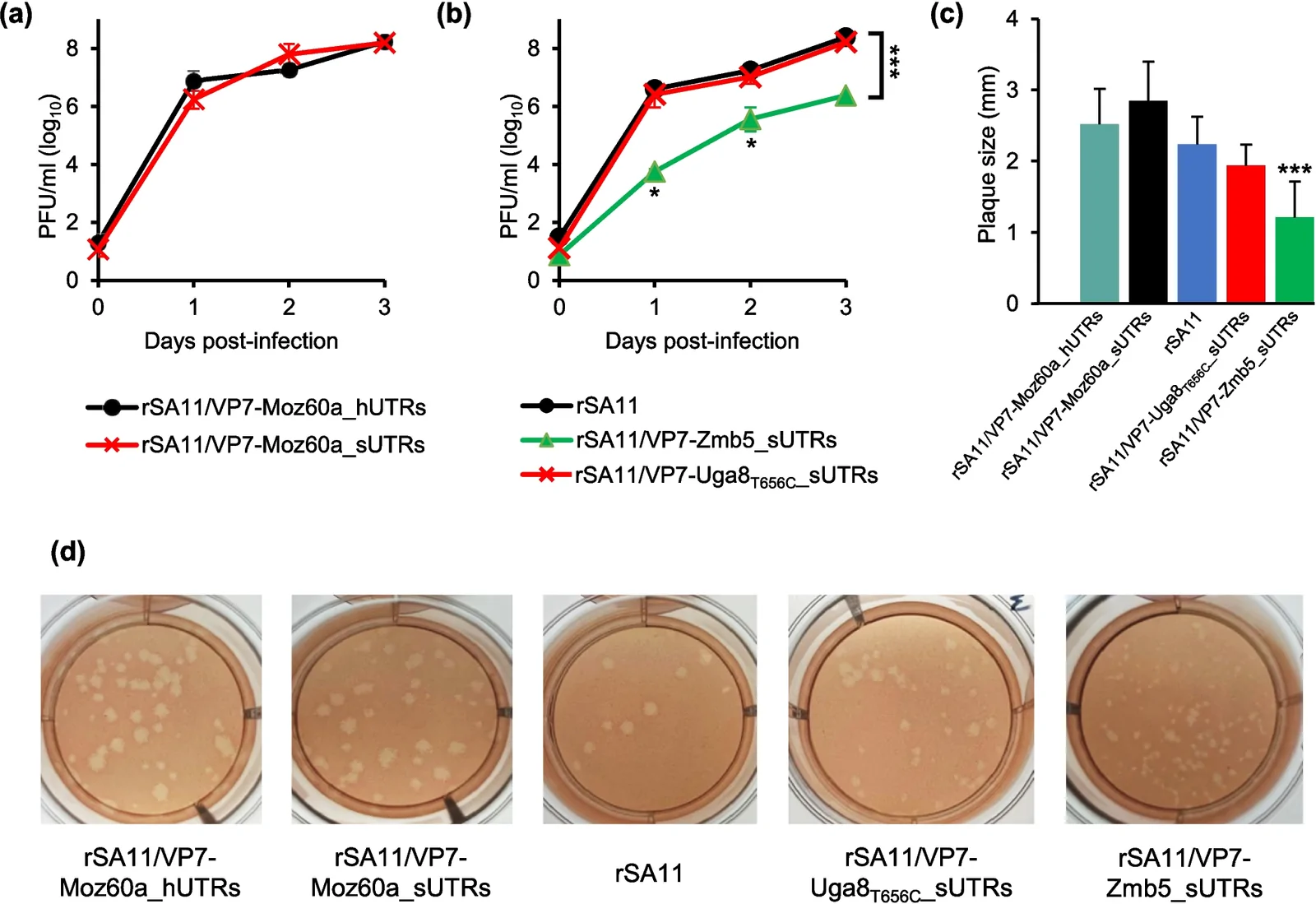
Characterization of selected reassortants by growth curves and plaque size **(a-b)** Analyses of replication kinetics. Cells were infected with a multiplicity of infection (MOI) of 0.003. Culture supernatants were collected and the number of PFU/mL was determined by plaque assay at the indicated time points post-infection. Data are means ± standard deviation from three independent experiments. **(a)** Replication of reassortants with the VP7-encoding ORF of a human rotavirus flanked by the original human rotavirus UTRs or SA11 UTRs. **(b)** Replication of the two novel reassortants with the VP7-encoding ORF from Zmb5 or Uga8 with mutation T656C flanked by SA11 UTRs. *p < 0.05 for rSA11/VP7-Zmb5_sUTRs versus rSA11; ***p < 0.001 for rSA11/VP7-Zmb5_sUTRs versus rSA11. **(c)** Plaque sizes of generated reassortants. Analysis was done at 2 days after infection of MA-104 cells. Data are means ± standard deviation from 10 randomly picked plaques. ***p < 0.001 for rSA11/VP7-Zmb5_sUTRs versus rSA11. **(d)** Representative plaque assay images from the experiment presented in (c). Abbreviations: PFU = Plaque forming units; rSA11/VP7-Moz60a_hUTRs = Reassortant with VP7-ORF and original UTRs from human RVA strain Moz60a in the backbone of SA11; rSA11/VP7-Moz60a_sUTRs, rSA11/VP7-Zmb5_sUTRs, rSA11/VP7-Uga8_T656__sUTRs = Reassortants with the VP7-ORF of the indicated human RVA strain flanked by SA11 UTRs in the backbone of SA11; rSA11 = Recombinant SA11; UTRs = Untranslated regions

First, the replication kinetics in MA-104 cells of the reassortant with the VP7-ORF of strain Moz60a flanked by SA11 UTRs was compared to that flanked by the original human RVA UTRs. As shown in Fig. 3a, the resulting growth curves were highly similar with no statistically significant titer differences at any tested time point, suggesting that the VP7-ORF of human RVA was highly compatible with the UTRs from simian RVA. Next, the replication of the two novel reassortants with the VP7-ORF from Zmb5 or Uga8_T656C_ was compared to rSA11. No titer differences in comparison to rSA11 were detected using the Uga8_T656C_ VP7-ORF (Fig. 3b). In contrast, the Zmb5 VP7-ORF reassortant replicated significantly slower, with the highest titer difference of approximately 3 logs at day 1 post-infection (Fig. 3b). The size of plaques induced by the reassortants were compared at day 2 after infection. In comparison to rSA11, the Zmb5 VP7-ORF reassortant induced significantly smaller plaques (Fig. 3c). Representative plaque assay images are shown in Fig. 3d.

## Discussion

Complete genome segment sequence information is generally required for the targeted generation of rotavirus reassortants utilizing reverse genetics approaches. However, the terminal regions of the rotavirus genome segments, which include the UTRs, are generally difficult to sequence. In most cases, additional sophisticated techniques like rapid amplification for cDNA ends (RACE) or primer ligation methods have to be applied for terminal sequence determination (Falkenhagen et al. 2022; Trojnar et al. 2009). Therefore, only the ORF sequences are determined and reported for many field strains, which hampers their direct inclusion as new antigens into vaccine strains by reverse genetics approaches. Here we showed that UTRs from simian RVA can be successfully used instead of the original UTRs, resulting in viable reassortants in most cases. By this, we were able to newly generate reassortants of human RVA VP7 G5 and G8 identified in Africa, thus broadening the antigenic repertoire for potential next generation RVA vaccine strains.

Primary and secondary UTR structures play important regulatory roles in the RVA replication cycle. The 5’ UTRs of several genome segments of SA11 carry the inhibitory motif 5’-GGY(U/A)UY-3’ which was shown to reduce viral protein levels (De Lorenzo et al. 2016). This inhibitory motif is also present in the UTR of the VP7-encoding genome segment of each strain with known UTR sequences in our study (Supplementary Fig. S1). Both 5’ and 3’ UTRs can also contain non-essential replication-enhancing cis-acting signals (Patton et al. 1996), while nucleotides at the end of the 3’ UTR serve as a polymerase recognition site and are essential for genome segment replication (Chen et al. 2001; Chen and Patton 1998; Lu et al. 2008; Patton et al. 1996; Silvestri et al. 2004; Tortorici et al. 2003). It has also been shown that the formation of the genomic RNA complex necessary for RVA genome packaging can be dependent on specific RNA–RNA interactions mediated by larger 3′-UTR sequences of specific genome segments (Fajardo et al. 2017). Furthermore, RVA RNAs fold into panhandles by interactions between 5’ and 3’ UTRs (Li et al. 2010; McDonald and Patton 2011; Tortorici et al. 2006), which contain important functional elements for assortment and packaging of RVA genome segments. However, it is not known to what extend sequence differences in UTRs from different RVA strains can influence their functionality. While nucleotide differences in the UTRs of the examined strains were identified in our study, they did not seem to have a negative effect on reassortment. A comparison of the replication kinetics of the Moz60a VP7-ORF reassortant with UTRs of simian RVA with that containing its original UTRs also confirmed that exchange of UTRs between strains had no obvious negative impact on viral replication.

The generation of two reassortants with VP7-ORFs from human RVAs (Uga8 and Caf29) was initially not successful. Detailed analyses of the deduced VP7 amino acid sequences identified the unusual substitution A219V in strain Uga8 and K28N in strain Caf29. In a previous study, we have shown that specific point mutations can substantially influence the generation of reassortants in cell culture (Valusenko-Mehrkens et al. 2024). Correction of the causative mutation in Uga8 eventually enabled the effective rescue of the corresponding reassortant. The VP7 protein consists of two tightly packed domains: a Rossmann-fold (domain I – residues 78–160 and 256–312) and a β-barrel (domain II – residues 161–255) which is integrated into one of the loops of the Rossmann-fold (Aoki et al. 2009)(Supplementary Fig. S3a). In mature virus particles, VP7 forms a trimer and is not only in contact with other VP7 molecules but also with VP4 and VP6. Mapping the residue at position 219 to the 3D structure of a VP7 trimer revealed that this residue is part of a jelly roll structure in the β-barrel domain and not located at a VP4 or VP6 contact site. However, residue 219 is adjacent to another VP7 molecule of the same trimer and a hydrogen bond is predicted to form between residue 220 and residue 291 of the adjacent VP7 molecule (Supplementary Fig. S3b). Protein structure analyses also showed that the sidechain of residue 219 in case of Ala is pointing towards the sidechain of Val225 and that a hydrogen bond is predicted between Ala219 and isoleucine (Ile) at position 193 (Supplementary Fig. S3b). Therefore, it might be speculated that A219V negatively affects VP7 inter- and/or intramolecular interactions rather than directly affecting the interaction with VP4 or VP6.

The sequence of strain Uga8 was originally determined by next-generation sequencing (Bwogi et al. 2017), a commonly used—but not error-free—method for genotyping in current RVA surveillance and monitoring programs (Khakha et al. 2023; Munlela et al. 2020; Strydom et al. 2019; Fox et al. 2014; Meaburn and Schulz 2012). It might therefore be speculated that the uncommon mutation was caused by a sequencing error. However, it could also represent a unique mutation originally present in this specific field strain, which only impeded its replication in cell culture. In either case, identification of unusual mutations followed by their correction by site-directed mutagenesis can represent a useful tool to recover infectious reassortants from previously reluctant strains.

In contrast, correction of the mutation resulting in K28N in strain Caf29 did not facilitate the generation of the corresponding reassortant. Residue 28 of the VP7 protein is located inside a signal peptide which is required for the translocation of VP7 to the endoplasmic reticulum, rapidly cleaved during synthesis, and not present in the mature protein (Stirzaker et al. 1990; Whitfeld et al. 1987). While K28N might affect intracellular sorting of VP7, our results suggest that the presence of this substitution was at least not the sole reason for the unsuccessful reassortant generation. We identified two additional amino acid residues at position 68 and position 73 of VP7 that were unique to Caf29 VP7, but did not test their effect on reassortant generation as residues with similar physico-chemical properties were present in other related strains. Nevertheless, these residues are part of the N-terminal VP7 arm (residues 51–77, Supplementary Fig. 3c) which interacts with VP4, VP6 and other VP7 molecules (Aoki et al. 2009; Chen et al. 2009; Settembre et al. 2011). Ser68 is also adjacent to the glycosylated residue Asn69 (Chen et al. 2009), and glycosylation of VP7 has been shown to facilitate correct protein folding (Mirazimi and Svensson 1998). In addition to the unsuccessful generation of the Caf29 reassortant, one of the newly generated reassortants (Zmb5) replicated less efficiently than the other tested reassortants. The reasons behind these differences are not known so far. Aside from potential sequencing errors, it can be speculated that VP7 of Caf29 and Zmb5 may not properly mediate infection in the tested cell culture system or may be incompatible with the SA11 backbone. Indeed, restrictions in reassortment of specific heterologous genome segments within the SA11 backbone leading to reduced or absent growth have been reported before (Falkenhagen et al. 2022; Mingo et al. 2017; Patzina-Mehling et al. 2020; Sanchez-Tacuba et al. 2020). Furthermore, some genotype constellations occur more frequently in nature than others, indicating favorable combinations (McDonald et al. 2009; Suzuki et al. 2024).

In conclusion, complete UTR sequence information is not necessarily required for the targeted generation of VP7-encoding genome segment reassortants using reverse genetics approaches. However, generation of recombinant reassortants may be impeded by potential sequencing errors or intrinsic reassortment limitations. The system established here could help to further broaden the antigenic repertoire for potential novel RVA vaccine strains.

## Supplementary Information

Below is the link to the electronic supplementary material.

Supplementary file1 (PDF 3173 KB)

## Data Availability

All data supporting the findings of this study are available within the manuscript and its Supplementary Information. Additional details are available upon reasonable request from the corresponding author.
