# Supplementary material for: Engineering human/simian rotavirus VP7 reassortants in the absence of UTR sequence information
Source: Appl Microbiol Biotechnol. 2025 Feb 27;109:52. doi: 10.1007/s00253-025-13435-z (PMC11868164; doi:10.1007/s00253-025-13435-z)
Supplement: Supplementary file 1 [file 253_2025_13435_MOESM1_ESM.pdf]

Applied Microbiology and Biotechnology

Supplementary Information for

Engineering human/simian rotavirus VP7 reassortants in the absence of UTR sequence information

Roman Valusenko-Mehrkens; Reimar Johne; Alexander Falkenhagen*

Department of Biological Safety, German Federal Institute for Risk Assessment, Max-Dohrn-Str. 8-10, 10589 Berlin, Germany

*Corresponding author
Email: alexander.falkenhagen@bfr.bund.de
Phone: +49-30-18412-24603

Table S1 Primers used for RT-PCR analysis, Sanger sequencing and mutagenesis. The bold letters mark the position of the C84G and T656C mutations that have been introduced into the VP7-ORF of Caf29 and Uga8, respectively.

Primer	Sequence	Figure
VP7-Plasmid Seq-F	5′-CGTTGTAAAACGACGGCCAG-3′	n/a
VP7-Plasmid Seq-R	5′-CGGCTCGTATGTTGTGTGGA-3′	n/a
VP7 Seq-F	5′-TAAACTGGAAAAAATGGTGGCA-3′	1c/2b
VP7 Seq-R	5′-GGTCACATCGAACAATTCTAAC-3′	1c/2b
VP7 Seq-R-Moz308	5′-GGTCACATCATACAAATCCGAC-3′	1c/2b
Caf29_mut_C84G-F	5′-CATTGAAATCAATAGTTAA**g**ATGATGGACTTTATTA-3′	2a
Caf29_mut_C84G-R	5′-TATAGCGTATTAAAAACAGCGATATCAACAAA-3′	2a
Uga8_mut_T656C-F	5′-GAAGAAATTG**c**AACAGCTGAGAAATTGG-3′	2a
Uga8_mut_T656C-R	5′-AAAAGTCGTAGTGTCAGTAGTAAGACATCC-3′	2a

n/a = not applicable

Table S2 Results of BLASTp search of the NCBI non-redundant protein sequence database using the complete amino acid sequence of VP7 of strain Uga8. Amino acid residues 211-230 of Uga8 and the five closest related sequences are shown. The bold letter denotes the amino acid residue at position 219. The strain designation and GenBank accession number are indicated. The references are listed where applicable.

Strain	GenBank accession number	Protein sequence (residues 211-230)	Reference
RVA/Human-wt/UGA/MUL-13-308/2013/G8P[6] (Uga8)	ASM56465	DTTTFEEI**V**TAEKLAITDVV	(Bwogi et al. 2017)
RVA/Human-wt/KEN/MRC-DPRU1606/2009/G8P[4]	AGV31633	DTTTFEEI**A**TAEKLAITDVV	(Nyaga et al. 2014)
RVA/Human-wt/RWA/UFS-NGS-MRC-DPRU656/2013/G8P[4]	WJN01071	DTTTFEEI**A**TAEKLAITDVV	(Mwangi et al. 2023)
RVA/Human-wt/KEN/KLF0600/2012/G8P[4]	QXY09342	DTTTFEEI**A**TAEKLAITDVV	(Mudibo 2021)
RVA/Human-wt/KEN/KLF0320/2010/G8P[4]	QXY08807	DTTTFEEI**A**TAEKLAITDVV	(Mudibo 2021)
Rotavirus A Hu/1290/Kenya/1991/G8	ACC96235	DTTTFEEI**A**TAEKLAITDVV	n/a

n/a = not applicable

Table S3 Results of BLASTp search of the NCBI non-redundant protein sequence database using the complete amino acid sequence of VP7 of strain Caf29. Amino acid residues 18-37 of Caf29 and the five closest related sequences are shown. The bold letter denotes the amino acid residue at position 28. The strain designation and GenBank accession number are indicated. The references are listed where applicable.

Strain	GenBank accession number	Protein sequence (residues 18-37)	Reference
RVA/Human-wt/CAF/CAR91/2014/G29P[6] (Caf29)	QKE60322	LIRYTLKSIV**N**MMDFIIYKF	(Banga-Mingo et al. 2021)
RVA/Buffalo-wt/ZAF/4426/2002/G29P[14]	QST88831	LIRYTLKSIV**K**MMDFIIYRF	(Strydom et al. 2020)
RVA/Human-wt/Bel/BEF06018/2014/G29P41	ANS11444	LIRYTLKSIV**K**MMDFIIYRF	n/a
Murine RVA isolate MelMuRV	ABM55469	LLRYVLKSVV**K**MMDFIVYRV	n/a
Murine rotavirus EL	AAA50490	LLRYILKSVV**K**MMDFIVYRF	(Dunn et al. 1994)
Simian RVA strain RRV	P12476	LLNYILKSLT**R**MMDFIIYRF	(Green et al. 1987)

n/a = not applicable

Table S4 Amino acid residues in VP7 unique to Uga8 and Caf29 in comparison to the 100 closest related strains.

Strain	Residue	Corresponding residue in the 100 closest related strains
Uga8	Val219	Ala
Caf29	Asn28	Arg, Lys
	Ser68	Ala, Thr, Val
	Thr73	Ala, Arg, Gln, Gly, Glu, Pro, Ser

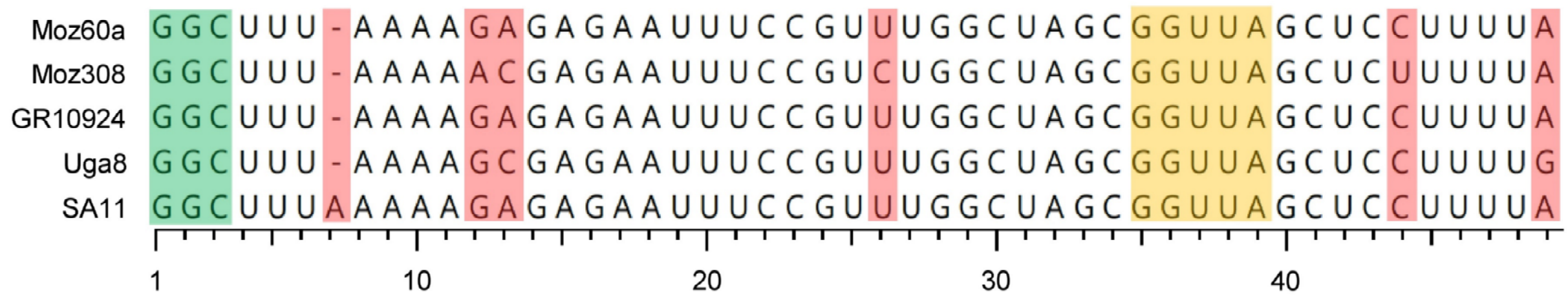

Fig. S1 5' UTR sequences of the VP7-encoding genome segment from the four human RVA strains Moz60a, Moz308, GR10924 and Uga8 and simian strain SA11. The alignment is a comparison between the 5' UTRs (nucleotides 1-49) of the indicated strains. The green square marks the highly conserved nucleotides at the start of the 5' UTR. Positions with sequence differences are marked in red. The orange square marks an inhibitory motif present in the 5' UTR that has been identified previously (De Lorenzo et al. 2016).

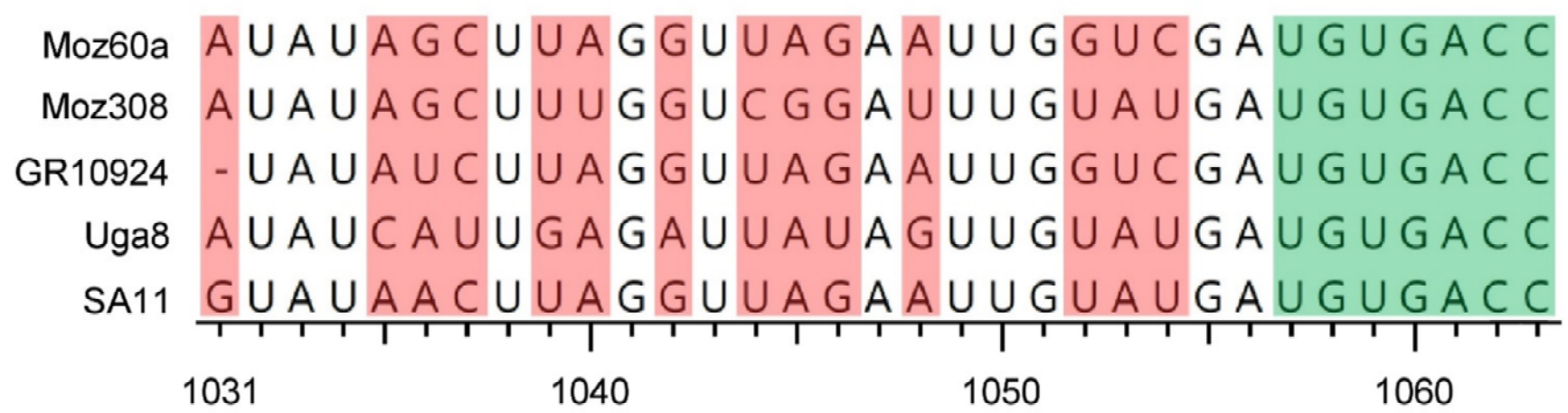

Fig. S2 3' UTR sequences of the VP7-encoding genome segment from the four human RVA strains Moz60a, Moz308, GR10924 and Uga8 and simian strain SA11. The alignment is a comparison between the 3' UTRs (nucleotides 1031-1063) of the indicated strains. The red squares mark positions with sequence differences. The green square marks the highly conserved nucleotides at the end of the 3' UTR.

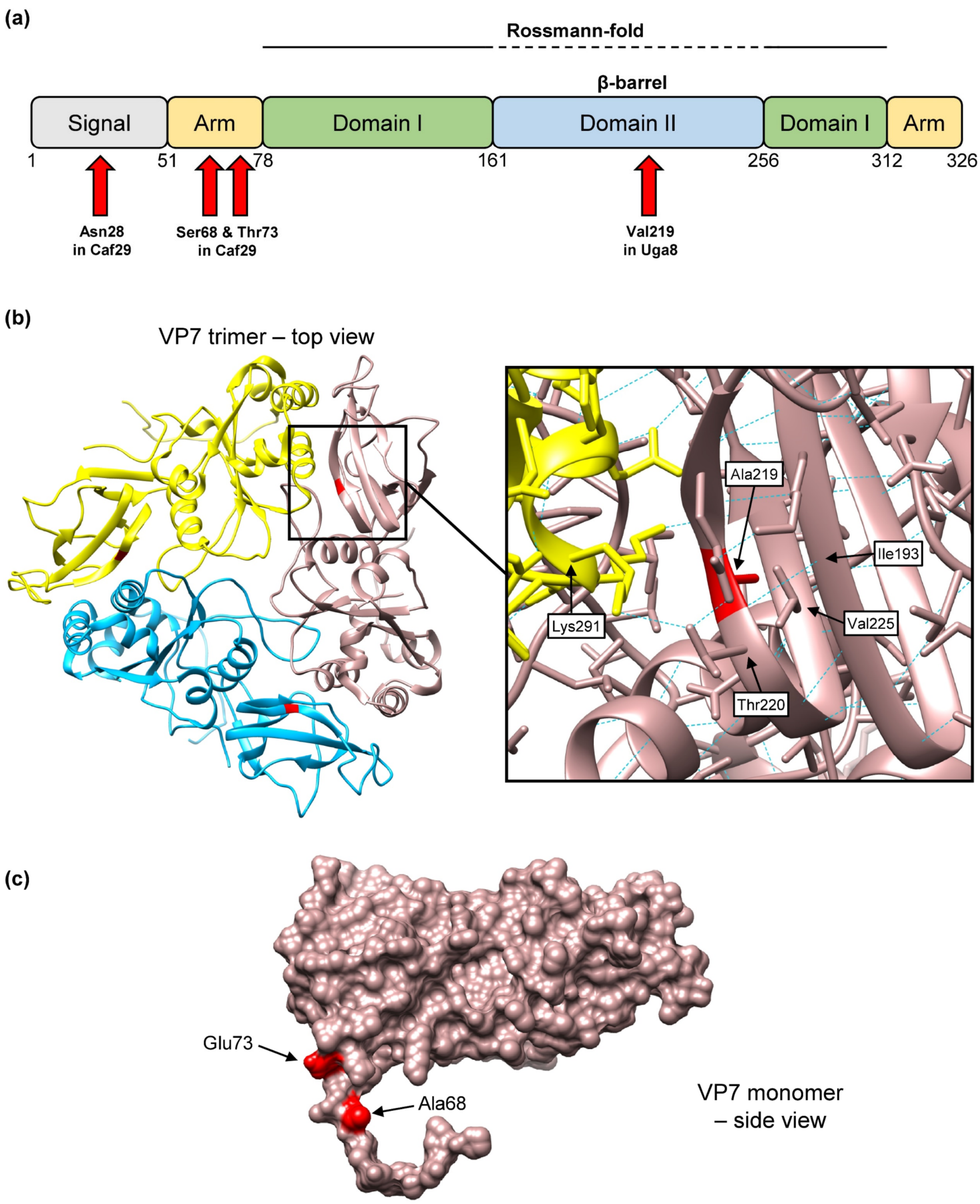

Fig. S3 Location of identified mutations in Uga8 and Caf29. **(a)** Schematic overview of VP7 and identified amino acid residues unique to Uga8 and Caf29 in comparison to related strains. Signal = Signal peptide cleaved in mature protein; Arm = N- and C-terminal extensions of the Rossmann-fold; Domain I = Rossmann-fold; Domain II = β-barrel domain inserted in the Rossman-fold; Red arrows = Unique amino acid residues. **(b)** Top view of the three-dimensional structure of a VP7 trimer based on the atomic model of infectious rhesus rotavirus (RRV) particle (PDB 4v7q, chain BL, BM and BN). Ala219 in RRV corresponding to Val219 in VP7 from Uga8 is highlighted in red. Hydrogen bonds are predicted between Ala219 and Ile193 as well as between Thr220 and Lys291 of an adjacent VP7 molecule of the same trimer. Predicted hydrogen bonds are depicted as dashed lines in cyan. The sidechains of Ala219 and Val225 are pointing toward each other. **(c)** Side view of the predicted surface structure of a VP7 monomer (PDB 4v7q, chain BM). Ala68 and Glu73 in RRV corresponding to Ser68 and Thr73 in Caf29 VP7, respectively, are highlighted in red.

References

Banga-Mingo V, Esona MD, Betrapally NS, Gautam R, Jaimes J, Katz E, Waku-Kouomou D, Bowen MD, Gouandjika-Vasilache I (2021) Whole gene analysis of a genotype G29P[6] human rotavirus strain identified in Central African Republic. BMC Res Notes 14(1):1-5 doi:10.1186/s13104-021-05634-4

Bwogi J, Jere KC, Karamagi C, Byarugaba DK, Namuwulya P, Baliraine FN, Desselberger U, Iturriza-Gomara M (2017) Whole genome analysis of selected human and animal rotaviruses identified in Uganda from 2012 to 2014 reveals complex genome reassortment events between human, bovine, caprine and porcine strains. PLoS One 12(6):1-23 doi:10.1371/journal.pone.0178855

De Lorenzo G, Drikic M, Papa G, Eichwald C, Burrone OR, Arnoldi F (2016) An inhibitory motif on the 5'UTR of several rotavirus genome segments affects protein expression and reverse genetics strategies. PLoS One 11(11):1-20 doi:10.1371/journal.pone.0166719

Dunn SJ, Burns JW, Cross TL, Vo PT, Ward RL, Bremont M, Greenberg HB (1994) Comparison of VP4 and VP7 of five murine rotavirus strains. Virology 203(2):250-259 doi:10.1006/viro.1994.1482

Green KY, Midthun K, Gorziglia M, Hoshino Y, Kapikian AZ, Chanock RM, Flores J (1987) Comparison of the amino acid sequences of the major neutralization protein of four human rotavirus serotypes. Virology 161(1):153-159 doi:10.1016/0042-6822(87)90181-4

Mudibo EO (2021) Understanding the sources of introductions and genetic diversity of rotavirus A strains through whole genome analysis of rotavirus G8P[4] and G9P[8] strains detected in Kilifi Kenya, 2010 - 2019. Master's Thesis, Pwani University

Mwangi PN, Potgieter RL, Uwimana J, Mutesa L, Muganga N, Murenzi D, Tusiyenge L, Mwenda JM, Mogotsi MT, Rakau K, Esona MD, Steele AD, Seheri ML, Nyaga MM (2023) The evolution of post-vaccine G8P[4] group A rotavirus strains in Rwanda; notable variance at the neutralization epitope sites. Pathogens 12(5):1-19 doi:10.3390/pathogens12050658

Nyaga MM, Stucker KM, Esona MD, Jere KC, Mwinyi B, Shonhai A, Tsolenyanu E, Mulindwa A, Chibumbya JN, Adolfine H, Halpin RA, Roy S, Stockwell TB, Berejena C, Seheri ML, Mwenda JM, Steele AD, Wentworth DE, Mphahlele MJ (2014) Whole-genome analyses of DS-1-like human G2P[4] and G8P[4] rotavirus strains from Eastern, Western and Southern Africa. Virus Genes 49(2):196-207 doi:10.1007/s11262-014-1091-7

Strydom A, Donato C, Peenze I, Potgieter AC, Seheri M, O'Neill HG (2020) Genetic characterisation of novel G29P[14] and G10P[11] rotavirus strains from African buffalo. Infect Genet Evol 85:1-6 doi:10.1016/j.meegid.2020.104463
